# A Remedial Institution

**Published:** 1875-09

**Authors:** 


### A REMEDIAL INSTITUTION.

We allude now to the establishment of the Drs. Strong, at Saratoga, widely known for the last 20 years as the "Saratoga Springs Remedial Institute." We alluded to it in our notes of travel last month, and expressed our intention to speak of the special methods employed in the treatment of diseases, at a future day. That promise we now partially redeem.

First, a word as to the location of the Institute. To our mind this is unexceptionable. Dr. Strong, the elder, who selected this spot for the practice of his profession, reasoned wisely when he argued that there were and ever would be great attractions at Saratoga, in its pure air and its mineral waters. He knew that invalids and semi-invalids would flock thither in search of health. He knew, too, that not all the waters of all the springs of the earth could wash out some diseases, unaided by other agencies. The waters would attract them, and he would cure them when the waters failed. So the Doctor set up his standard on an elevated site near the heart of the town, and here he has flourished, expanded and grown great. That he has done much good, is the testimony of all at Saratoga, and that he has been the means of saving a greater number of lives in two decades than the springs have saved this century, it is reasonable to assume.

For some years past, Dr. Strong has had the valuable aid of his son. He, too, is a medical graduate, and his experience—not only at the Institute, but in hospital and army service—has been very great.

The theory of the institute is one which every intelligent physician or layman must endorse. It is the theory of assisting and encouraging nature. The old method was to first clean out and then build up. The blood was cleaned by puncturing a vein and allowing it run out. The less blood there was the less *bad* blood there was. Another favorite plan was to give a powerful emetic or a strong catharic,—to clean things out,—before proceeding further. The fact that nature was all the time engaged in the most effective cleansing processes, was ignored, and the poor bodies were racked with needless and injurious torture. But the lives of our sick folk of to-day, have fallen in pleasanter places. The thoughtful physician finds success in a thousand ways, without indulging in the destructive heroics of the barbarous past. So it is with the Drs. Strong. They have learned that there is a whole armory of weapons of more celestial temper, and more potent to heal the sick, than pills and powders and tinctures. These last they do not altogether discard, but they are not confined to them. They place great reliance in baths. So they have fitted up the Institute with a wonderful array of these appliances. They have a Turkish bath, with all the delights of perspiration and shampooing; a Russian bath, with steam so dense that it covers you as with a mantle; a sulphur bath, an electro-thermal bath—in short, all the baths which have been successfully employed in diseased states. They employ also the "Vacuum treatment"—by which the air is exhausted from a portion or the whole of the body—electricity, oxygen-gas, the hydropathic use of water—and all the adjuncts of the best hospitals. It is not strange, then, that their success is great, and that the Institute is well patronized the year round, even when the mere hotels of the famous watering-place are silent and deserted.

---

If rats are about, scatter powdered glass about their holes, or powdered copperas, or fill up the crevices with hard soap, or smear their holes with soft tar, or dip the rat in a cup of tar and let it go, and it will tar-plaster every hole in the house.

---

Common lye of wood ashes will soften hard putty in a few minutes.

